# An Otogenic Trapezius Abscess: A Case Report

**Published:** 2012

**Authors:** Fazal I Wahid, Adil Khan, Iftikhar Ahmad Khan

**Affiliations:** 1 *Department of ENT, Head and Neck Surgery, Postgraduate Medical Institute, Lady Reading Hospital, Peshawar, Pakistan.*

**Keywords:** Complications of chronic suppurative otitis media, Trapezius abscess, Rare abscess

## Abstract

**Introduction:**

An otogenic brain abscess is a common ENT problem but an otogenic trapezius abscess can also be experienced in otolaryngological practice, particularly in patients with chronic suppurative otitis media.

**Case Report:**

We report a rare case of a trapezius abscess in an eight-year-old girl who presented at the ENT, Head and Neck Surgery Postgraduate Medical Institute, Lady Reading Hospital, Peshawar, Pakistan on 15th December, 2010, with a presenting complaint of discharge from her right ear that had been occurring for the last five years. An exploration of the patient’s right ear was performed, which showed that there was extensive cholesteatoma and tissue granulation tissues the antrum, attic and middle ear. The trapezius abscess had spread down to her back and was repeatedly drained. The patient was discharged on the 14th day following admission after making a complete recovery. After a regular follow-up period the child has remained disease free. The rare nature of this case prompted us to write this report.

**Conclusion:**

Chronic suppurative otitis media is a common clinical problem in developing countries. It can result in a number of complications if not treated properly. Although an otogenic trapezius abscess is a rare complication of chronic suppurative otitis media, it must be kept in mind.

## Introduction

The principal aims of a tympanoplasty Chronic suppurative otitis media (CSOM) is an inflammatory process within the middle ear cleft, which is associated with irreversible tissue pathology and characterized by a persistent discharge from the middle ear through a tympanic perforation. CSOM is a dangerous illness (1). In spite of a significant decrease in cases of CSOM following the advent of antibiotics, complications of otitis media still represent a challenging situation owing to their high mortality rate. Complications can be classified as intra temporal or intra cranial, which include extradural abscesses, brain abscesses, subdural abscesses, sigmoid sinus thrombophlebitis, otic hydrocephalus and meningitis, or extra cranial, which includes acute mastoiditis, mastoid abscesses, petrositis, labyrinthitis, facial paralysis and osteomyelitis of the temporal bone (2). Factors that can cause complications include the level of virulence of the infectious organism, poor resistance of the patient, the presence of chronic systemic diseases and resistance of the infecting organism to antibiotics (3). 

Otogenic abscesses only develop in patients with chronic otitis media, and 90% of those patients also have cholesteatoma. This trend has been observed by others; Nunes and Browning reported that 95% of otogenic abscesses were found in patients with chronic otitis media and 41% of those patients were found to have cholesteatoma (4). The contemporary risk for developing extracranial complications of otitis media is approximately twice that for developing intracranial complications, with 0.45% of patients with otitis media experiencing problems such as a subperiosteal abscess (5). Inflammation and infection may result in necrosis of the mastoid tip, allowing the pus to track from the medial side of the mastoid process through the incisura digastrica (digastric groove). The pus is prevented from reaching the body surface by the neck musculature, but can track along the fascial planes of the digastic muscle, sternomastoid or trapezius muscles. Pneumatization of the mastoid process leads to thinning of the bone and is considered an important factor in the development of a trapezius or Bezold’s abscess. Therefore, these abscesses are more common in adults than in children because the mastoid tip is often well pneumatized in adults (6).

## Case report

Here we report a rare case of a trapezius abscess. An eight-year-old girl presented at the Out-Patient Department of the ENT, Head and Neck Surgery Postgraduate Medical Institute, Lady Reading Hospital, Peshawar, Pakistan on 15th December, 2010, with a complaint of discharge from the right ear that had been occurring for the last five years. The discharge was scant and foul smelling and was associated with high grade fever. The patient had developed swelling in the right postauricular area 15 days before admission to hospital. She belonged to a poor family. Her past, personal, medical and allergic history was unremarkable. 

**Fig 1:**
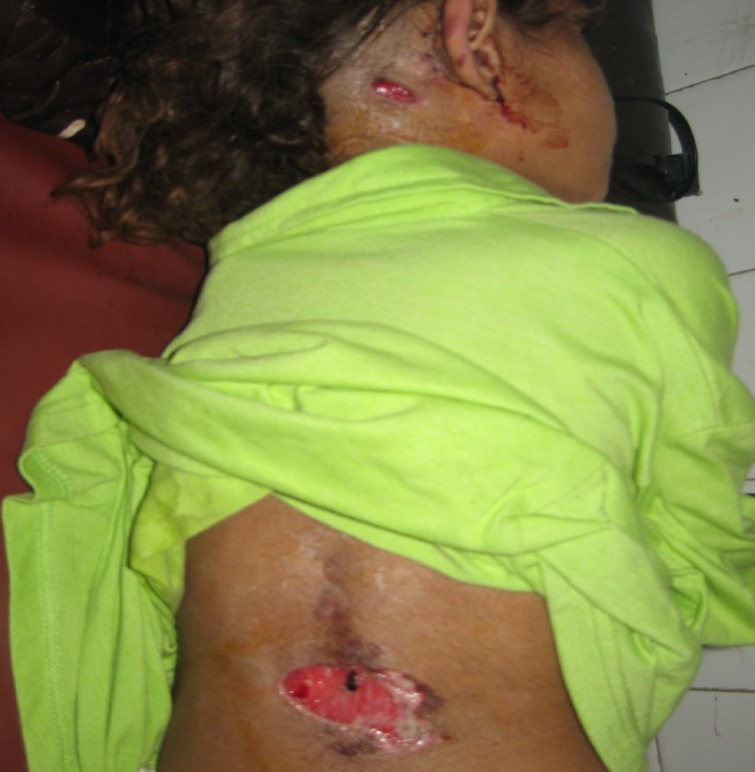
Post-operative photograph of the patient showing the right mastoid abscess and how the trapezius abscess had spread down to the back. The two incisions show the mastoid and trapezius abscesses drained

**Fig 2:**
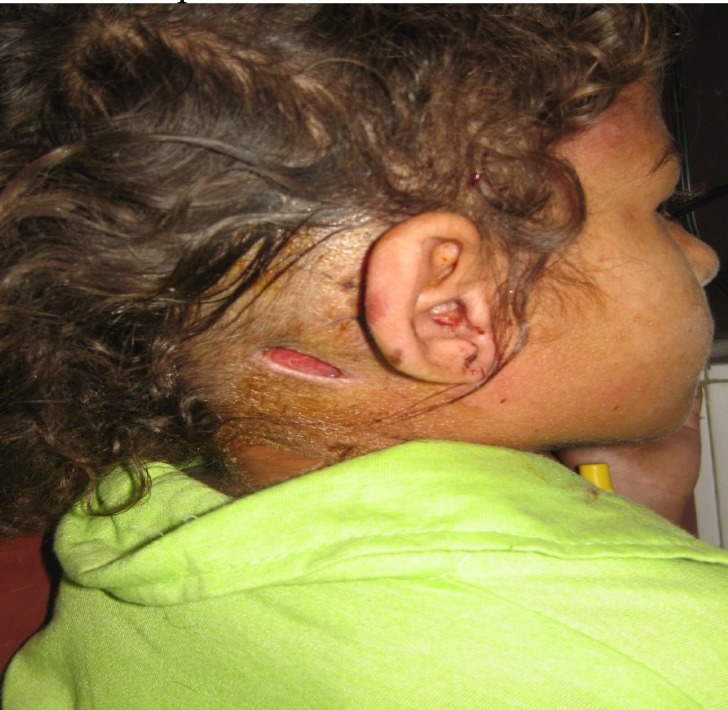
Post-operative photograph of the patient showing the radical mastoidectomy in the  right ear

On examination there was an atticoantral-type perforation of the right tympanic membrane with a tender, tense abscess in the right postauricular area (Fig. 1). A similar swelling was found on the patient’s back (Fig. 2). The mastoid abscess was drained and the patient was put on an injectable quadruple antibiotic regimen constituting benzyl penicillin, gentamycin, chloromycetin and metronidazole. She was closely monitored but there was no improvement in her condition after 48 hours. A CT scan of the temporal bone and brain was performed and intracranial complications were excluded. On the third day following admission to hospital, an exploration of the patient’s right ear was performed and a radical mastoidectomy was carried out on the right side where there was extensive cholesteatoma and tissue granulation involving the antrum, attic and middle ear. It was also observed that the pus from the trapezius abscess had spread down to the back along the trapezius muscle to form an abscess on the lower back. The abscess was repeatedly drained due to recurrence of the abscess as pus trickled down from the mastoid region. The reason for the continued spread of pus was a well pneumatized mastoid tip and poor patient response to medication. She received a two-week course of antibiotics and was then discharged following a complete recovery.

## Discussion

The ancient Egyptians recognized CSOM as a disease of the ear and treated it with duck grease, borax, and cow milk. Hippocrates understood the recurrent nature of CSOM and placed patients on different medical and behavioral therapies, depending on the time course of their suppuration (7). Despite the tremendous advances made in the treatment of all forms of otitis media, complications still occur and often represent the most life-threatening conditions, requiring immediate and precise therapeutic intervention (8). For most otolaryngologists dealing with CSOM, a high index of suspicion and prompt management of patients with intracranial complications is the difference between life and death. In considering the different therapeutic options, the physician must be aware of the complications that can arise (9). CSOM can cause chronic mastoiditis, which initially affects the small bony ridges in the mastoid. If not properly treated, the inflammation can penetrate through the mastoid cortical bone behind the ear; even worse, it can enter the intracranial space and cause complications (10). In general, treatment of such complications consists of intravenous antibiotics. Surgical drainage is made in those cases that do not progress well. 

Extracranial abscesses secondary to CSOM are a disease of the indigent, and are prevalent in developing countries (11). The abscesses occur predominantly in children and young adults, with preponderance in males. Early and aggressive surgical interventions for otogenic abscesses should result in a minimal morbidity. Delay in surgery will lead to clinical worsening and poor results. Studies show that patients operated on within 72 hours have a 10% rate of disability compared with a 70% rate if the surgery took place after 72 hours (12). Surgical drainage and/or incision along with aggressive antibiotic therapy remains the definitive treatment for otogenic abscesses. Some studies utilizing aspiration as the only surgical intervention have produced excellent results and this has also been advocated as a first line therapy. Conservative therapy alone is recommended in selected cases, such as in the early stages of an abscess. However, surgical treatment along with antibiotics is the preferred regimen nowadays, which helps in reducing mortality and was adopted in this case (13). The patient was put on a quadruple regimen of antibiotics, and when the acute stage of the disease was overcome the primary source of the disease was treated properly by performing radical masoidectomy on the right side. If CSOM is accompanied by complications it should be treated adequately and both the complications and the primary focus of the disease must be addressed properly to get a good outcome. 

## Conclusion

CSOM is a common clinical problem in developing countries. The condition can result in a number of complications if not treated properly, and although an otogenic trapezius abscess is a rare complication of CSOM it must be kept in mind.
